# Glypican-3 promotes cell proliferation and tumorigenesis through up-regulation of β-catenin expression in lung squamous cell carcinoma

**DOI:** 10.1042/BSR20181147

**Published:** 2019-06-25

**Authors:** Dongchang Wang, Yan Gao, Yu Zhang, Lifei Wang, Gang Chen

**Affiliations:** 1 Department of Respiration, The Third Hospital of Hebei Medical University, Shijiazhuang 050051, Hebei, China; 2 Department of General Medicine, China-Japan Friendship Hospital, Beijing 100029, China

**Keywords:** β-catenin, GPC3, lung squamous cell carcinoma, proliferation, tumorigenesis

## Abstract

As a cell surface proteoglycan anchored by glycosyl-phosphatidylinositol, Glypican-3 (GPC3) is reported to be highly expressed in hepatocellular carcinoma (HCC) and to promote cell proliferation and tumorigenesis through activating Wnt/β-catenin signalling. GPC3 is also overexpressed in lung squamous cell carcinoma (SCC), but its effects and mechanisms in the progression of lung SCC remain unknown. The present study aims to explore the role and molecular mechanism of GPC3 in the occurrence and development of lung SCC. Immunohistochemistry, Western blot (WB) and real-time PCR (RT-PCR) assays were used to determine the expression patterns of GPC3 in lung SCC tissues and cells. MTT, flow cytometry and *in vivo* xenotransplantation assays were used to evaluate the influence of GPC3 on the growth, apoptosis and tumorigenesis of lung SCC cells. The results showed that GPC3 expression levels in lung SCC tissues and cells were significantly elevated, and the high expression of GPC3 significantly promoted cell growth and tumorigenesis and repressed cell apoptosis, as well as increased β-catenin expression. Moreover, knockdown of β-catenin obviously weakened GPC3 role in the promotion of cell proliferation and tumorigenesis, as well as the inhibition of cell apoptosis. In conclusion, the present study demonstrates that up-regulation of GPC3 accelerates the progression of lung SCC in a β-catenin-dependent manner. Our study provides a theoretical basis for GPC3/β-catenin as a novel diagnostic marker and therapeutic target for lung SCC.

## Introduction

Lung cancer is one of the most frequently diagnosed malignancies and is the leading cause of cancer-related deaths in the world [1]. Non-small cell lung cancer (NSCLC) comprises approximately 85% of all types of lung cancer cases, among which lung adenocarcinoma (AC) and lung squamous cell carcinoma (SCC) are the two most common histologic subtypes [2]. In detail, SCC accounts for approximately 35% of NSCLC cases and causes an estimated 0.4 million deaths every year worldwide [3]. Increasing evidence has identified that low expression of tumour suppressor genes and/or hyper-activation of oncogenes obviously accelerates the occurrence and development of lung SCC [4–7]. Therefore, it is urgent to clarify the molecular mechanisms underlying lung SCC progression, hoping to provide potent insights into the treatment of lung cancer.

Glypican-3 (GPC3) is a cell surface proteoglycan anchored by glycosyl-phosphatidylinositol, which was first discovered in patients with Simpson–Golabi–Behmel syndrome [8]. GPC3 serves as a membrane co-receptor for heparin-binding growth factors, including Hedgehog (Hh) proteins, Wnts and fibroblast growth factors, thereby modulating cell growth, apoptosis and differentiation [9,10]. It was recently reported that GPC3 was overexpressed in the serum samples of hepatocellular carcinoma (HCC) patients compared with that in patients with liver cirrhosis or chronic hepatitis or healthy donors [11]. Moreover, overexpression of GPC3 significantly enhanced HCC cell viability [9], indicating the vital role of GPC3 in carcinogenesis. In addition, Aviel-Ronen et al. [12] explored the expression pattern of GPC3 in lung cancer for the first time and demonstrated that GPC3 was positively expressed in 55% of SCC versus 8% of adjacent cancer but negatively expressed in normal lung tissues, suggesting that the overexpression of GPC3 might play a role in the progression of lung SCC.

Capurro et al. [9] reported that overexpression of GPC3 promoted the activation of β-catenin. However, whether GPC3 induces β-catenin signalling activation in lung SCC remains unknown. In the present study, we aimed to investigate the function of GPC3 in the development and progression of lung SCC and to determine whether Wnt/β-catenin signalling is involved.

## Materials and methods

### Tissue samples

Thirty paired lung SCC tissues and paracancerous tissues acquired from patients with primary lung SCC who underwent surgical resection in the Third Hospital of Hebei Medical University between January 2015 and September 2017 were included in our study. All tissue samples were collected instantly after surgery and stored in liquid nitrogen for further experiments. The experimental work with human samples was carried out in accordance with the World Medical Association Declaration of Helsinki, and all subjects signed informed consents.

### Immunohistochemistry staining

All tissue samples were fixed in 10% formalin, embedded in paraffin and cut into 4-μm-thick slides for immunohistochemistry staining. Following antigen retrieval with citrate buffer (pH 6.0) at 100°C for 20 min, slides were incubated with 3% BSA diluted with PBS at room temperature for 1 h and subsequently incubated with anti-GPC3 antibody (1:150 dilution; No. ab66596, Abcam, CA, U.S.A.) overnight at 4°C. Then, the slides were incubated with horseradish peroxidase (HRP)–conjugated secondary antibody at room temperature for 1 h, followed by incubation with 3,3′-diaminobenzidine chromogenic reagent and Haematoxylin counterstaining.

### Cell culture

The human lung SCC cell lines NCI-H520, NCI-H226 and SK-MES-1, as well as the human normal lung cell line BEAS-2B, were all purchased from the American Type Culture Collection (VA, U.S.A.); the human lung SCC cell line LTEP-s was purchased from BeNa Culture Collection (Beijing, China). For cell culture, LTEP-s cells were cultured in DMEM with high glucose (Gibco, CA, U.S.A.); NCI-H520 and NCI-H226 cells were cultured in RPMI-1640 medium (Gibco, CA, U.S.A.); SK-MES-1 cells were cultured in MEM (Gibco, CA, U.S.A.), all with 10% foetal bovine serum (FBS; Gibco, CA, U.S.A.) and 1% penicillin and streptomycin supplement. All cells were maintained in a humidified incubator at 37°C with 5% CO_2_.

### GPC3/β-catenin up- and/or down-regulation

Lentiviruses containing GPC3-overexpressing plasmids (with neomycin resistance) and shRNAs targeting GPC3 (sh-GPC3; with neomycin resistance) and β-catenin genes (sh-β-catenin; with puromycin resistance), as well as their negative control (NC) nucleotide sequences, were all designed and synthesised by GenePharma (Shanghai, China). The online software (https://rnaidesigner.thermofisher.com/rnaiexpress/) was used to design the shRNA. The sequences with high score were selected and the knockdown efficiency was verified by real-time PCR (RT-PCR) and immunofluorescence technologies. To establish stable expressing cell lines, 400 μg/ml G418 and/or puromycin (Life Technologies) was added to the culture medium 48 h after transfection. After 7 days of G418 and/or puromycin treatment, cells were trypsinised and grown in a 96-well dish and expanded. shRNA sequences targeting human GPC3 and β-catenin genes are listed as follows:

**GPC3:**

sh1- Top strand 5′-CACCGCCAGGATCAGATTTGCAAGTCGAAACTTGCAAATCTGATCCTGGC-3′, sh1- Bottom strand 5′-AAAAGCCAGGATCAGATTTGCAAGTTTCGACTTGCAAATCTGATCCTGGC-3′;

sh2- Top Strand 5′-CACCGGACGCCACCTGTCACCAAGTCGAAACTTGGTGACAGGTGGCGTCC-3′, sh2- Bottom strand 5′-AAAAGGACGCCACCTGTCACCAAGTTTCGACTTGGTGACAGGTGGCGTCC-3′;

sh3- Top strand 5′-CACCGGATCAGATTTGCAAGTATGTCGAAACATACTTGCAAATCTGATCC-3′, sh3- Bottom strand 5′-AAAAGGATCAGATTTGCAAGTATGTTTCGACATACTTGCAAATCTGATCC-3′.

**β-catenin:**

sh1- Top strand: 5′-CACCGGATGTGGATACCTCCCAAGTCGAAACTTGGGAGGTATCCACATCC-3′, sh1- Bottom strand 5′-AAAAGGATGTGGATACCTCCCAAGTTTCGACTTGGGAGGTATCCACATCC-3′;

sh2- Top strand 5′-CACCGGTTAATAAGGCTGCAGTTATCGAAATAACTGCAGCCTTATTAACC-3′, sh2- Bottom strand 5′-AAAAGGTTAATAAGGCTGCAGTTATTTCGATAACTGCAGCCTTATTAACC-3′;

sh3- Top strand 5′-CACCGCATAACCTTTCCCATCATCGCGAACGATGATGGGAAAGGTTATGC-3′, sh3- Bottom strand 5′-AAAAGCATAACCTTTCCCATCATCGTTCGCGATGATGGGAAAGGTTATGC-3′.

### Western blot analysis

Total proteins were obtained from cells and tissues using cold RIPA buffer. After quantification with the BCA kit, proteins were subjected to sodium dodecyl sulphate/polyacrylamide gel electrophoresis, with each lane containing 20 μg of protein, and transferred to polyvinylidene difluoride membranes (PVDF; Millipore, CA, U.S.A.). Then, the membranes were sealed with 5% non-fat milk and incubated with primary antibodies against PI3K (No. #4292, Cell Signaling Technology, CA, U.S.A.), β-catenin (No. #9562, Cell Signaling Technology, CA, U.S.A.), AKT1 (No. #2920, Cell Signaling Technology, CA, U.S.A.), Wnt (No. #2391, Cell Signaling Technology, CA, U.S.A.), STAT1 (No. ab31369, Abcam, CA, U.S.A.) or GAPDH (No. #5174, Cell Signaling Technology, CA, U.S.A.) overnight at 4°C, followed by probing with HRP–conjugated secondary antibodies (Zhongshan Golden Bridge Biotechnology, Beijing, China) at 37°C for 1 h. After washing three times with PBS, the membranes were incubated with enhanced chemiluminescence reagents (Millipore, U.S.A.). For quantification, the Western blotting bands were quantified by ImageJ software (National Institutes of Health) after background subtraction. The protein expression level was normalised to GAPDH expression.

### RT-PCR

Total RNA from lung SCC cells or tissue samples was extracted using TRIzol reagent (Invitrogen, U.S.A.) according to the manufacturer’s description. Then, a total of 1 μg of total RNA from each sample was used to form the first-strand cDNA with M-MLV Reverse Transcriptase (Promega, U.S.A.), and RT-PCR was performed using SYBR Premix ExTaqII (Tli RNaseH Plus) (Takara, Dalian, China) on the ABI 7500 Real-time PCR Instrument (Life Technologies, CA, U.S.A.) in a 20-μl system. The relative expression of mRNA was calculated by the 2^−ΔΔ*C*t^ method. Primers used in the present study were provided by Beijing Genomics Institute (Beijing, China) and listed as follows:

**GPC3:**

Forward (F): 5′-TCATGCAAGGCTGTATGGCA-3′,

Reverse (R): 5′-TGCCAATCTGTAAGTCTAGCCC-3′;

**Wnt1:**

(F): 5′-CAAGATCGTCAACCGAGGCT-3′,

(R): 5′-AAGGTTCATGAGGAAGCGCA-3′;

**β-catenin:**

(F): 5′-GCGCCATTTTAAGCCTCTCG-3′,

(R): 5′-GGCCATGTCCAACTCCATCA-3′;

**PI3K:**

(F): 5′-CCAGGGAAATTCTGGGCTCC-3′,

(R): 5′-TGTATTCAGTTCAATTGCAGAAGGA-3′;

**AKT1:**

(F): 5′-CCAGCCTGGGTCAAAGAAGT-3′,

(R): 5′-TGTACTCCCCTCGTTTGTGC-3′;

**STAT1:**

(F): 5′-CACAAGGTGGCAGGATGTCT-3′,

(R): 5′-TCCCCGACTGAGCCTGATTA-3′;

**GAPDH:**

(F): 5′-AAAGCCTGCCGGTGACTAAC-3′;

(R): 5′-GACTCCACGACGTACTCAGC-3′.

### Cell proliferation assay

A total of 2×10^4^ LTEP-s/SK-MES-1 cells transfected with sh-GPC3 or sh-NC, vector-GPC3, vector-NC, or vector-NC + sh-NC, vector-GPC3 + sh-NC, vector-NC + sh-β-catenin, vector-GPC3 + sh-β-catenin were suspended with 100 μl of complete culture medium and then seeded into 96-well plates. After 24, 48, 72 or 96 h of culture, 10 μl of MTT (5 mg/ml; Sigma–Aldrich, MO, U.S.A.) solution was added to each well and incubated for an additional 4 h at 37°C in the dark. Subsequently, the medium was replaced with 100 μl of DMSO to dissolve the formazan. The absorbance of each well was measured at 570 nm using a microplate reader (Bio-Rad 550, CA, U.S.A.) to evaluate cell viability.

### Cell apoptosis assay

LTEP-s/SK-MES-1 cells were grown in 24-well plates overnight, and then different treatments, including sh-GPC3, sh-NC, vector-GPC3, vector-NC, vector-NC+sh-NC, vector-GPC3+sh-NC, vector-NC+sh-β-catenin, and vector-GPC3+sh-β-catenin, were given to these cells. After 48 h of treatment, cells were collected and submitted to apoptosis detection with the Annexin V (FITC)/propidium iodide (PI) Apoptosis Detection Kit (BD Biosciences, U.S.A.) according to the manufacturer’s instructions.

### Immunofluorescence

Transfected LTEP-s and SK-MES-1 cells were plated on glass coverslips in a 24-well dish at a density of 2×10^5^ cells/well and cultured for 48 h. Then, the cells were fixed with paraformaldehyde for 15 min at room temperature and incubated with rabbit polyclonal GPC3 antibody (No. ab66596, Abcam, MA, U.S.A.) or rabbit polyclonal β-catenin antibody (No. ab16051, Abcam, MA, U.S.A.), followed by the corresponding fluorescent secondary antibodies (Abcam, MA, U.S.A.). Nuclear DNA was labelled in blue with DAPI at a 1:10000 dilution (Solarbio, Beijing, China). Then, the glass coverslips were sealed with antifade reagent (Vectashield, Loerrach, Germany) and submitted to a laser scanning microscope (TCSSP2-AOBS-MP, Leica Microsystems CMS) with a magnification of 400×.

### *In vivo* tumorigenesis assay

Male BALB/c nude mice weighing 18–20 g purchased from Shanghai Biomodel Organism Science & Technology Development Co., Ltd. (Shanghai, China) were used for the *in vivo* tumorigenesis assay. Mice were fed in specific pathogen-free (SPF) conditions with standard food and water and submitted to a 12-h light/dark cycle. To explore the function of GPC3/β-catenin in the tumorigenesis of lung SCC cells, LTEP-s or SK-MES-1 cells (1 × 10^7^ cells diluted in 200 μl of PBS) with stable expression of vector-NC+sh-NC, vector-GPC3+sh-NC, vector-NC+sh-β-catenin or vector-GPC3 + sh-β-catenin were subcutaneously injected into nude mice, with five mice in each group. All mice were killed after 21 days of implantation, and tumours were removed and weighed. All protocols involving animals were carried out in accordance with the Animal Experiment Ethics Review of the Third Hospital of Hebei Medical University.

### Data analysis

All experiments were performed at least three times, and the data are presented as the mean ± standard deviation (SD). Differences between the experimental groups were assessed by Student’s *t* test using SPSS 19.0 software. *P*-values less than 0.05 were regarded as statistically significant.

## Results

### GPC3 is overexpressed in lung SCC tissues

**Figure 1:**
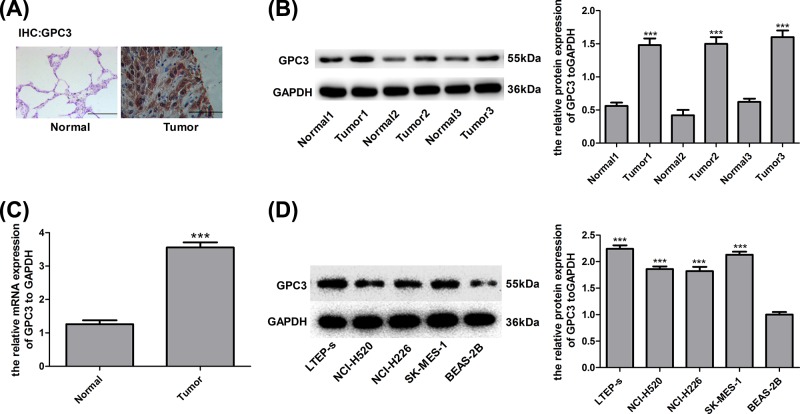
GPC3 was highly expressed in lung SCC cells and tissues (**A**) Immunohistochemistry assay with GPC3 staining was carried out to detect the expression of GPC3 in 30 paired lung SCC tissues and the adjacent normal tissues. A representative image is shown (scale bar = 100 μm). (**B**) WB was used to determine the protein expression of GPC3 in 30 paired lung SCC tissue samples and non-tumour tissue samples, and representative images are shown (***P<0.001, tumour group vs normal group). (**C**) RT-PCR analysis of the mRNA level of GPC3 in 30 paired lung SCC tissue samples and non-tumour tissue samples (***P<0.001, tumour group vs normal group). (**D**) WB analysis of GPC3 expression in the normal lung cell line BEAS-2B and lung SCC cell lines LTEP-s, NCI-H520, NCI-H226 and SK-MES-1 (***P<0.001, compared with the BEAS-2B group).

To explore the function of GPC3 in the occurrence and development of lung SCC, we first compared GPC3 expression patterns in lung SCC tissue samples and matched non-tumour tissues through immunohistochemistry, Western blot (WB) and RT-PCR. The results demonstrated that GPC3 expression at both the protein and mRNA levels was elevated in lung SCC tissues compared with adjacent non-tumour tissues (Figure 1A–C). In addition, we tested the expression of GPC3 in the normal lung cell line BEAS-2B and lung SCC cell lines including LTEP-s, NCI-H520, NCI-H226 and SK-MES-1. As shown from the WB results, the expression of GPC3 protein in NCI-H520, NCI-H226 and SK-MES-1 cells was obviously higher than that in BEAS-2B cells (Figure 1D). These results imply that GPC3 overexpression might play a role in the progression of lung SCC.

### Overexpression of GPC3 promotes the proliferation and inhibits the apoptosis of lung SCC cells

**Figure 2:**
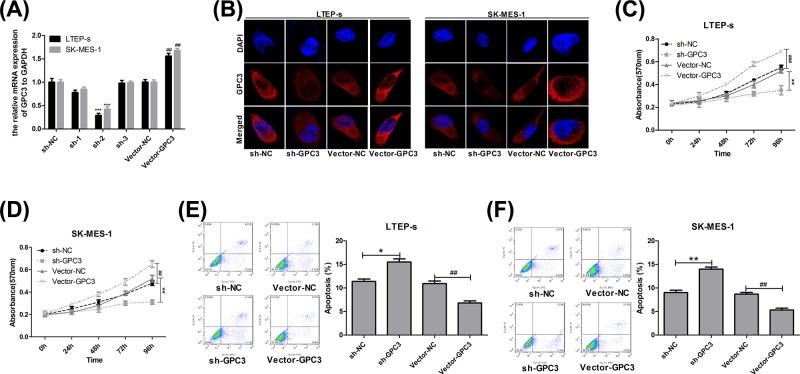
Evaluation of GPC3 effects on the proliferation and apoptosis of lung SCC cells (**A**) RT-PCR analysis of the mRNA level of GPC3 after LTEP-s and SK-MES-1 cells were infected with vector-GPC3, vector-NC, sh-GPC3 or sh-GPC3 for 48 h. (**B**) Immunofluorescence assay was used to detect the subcellular location and expression of GPC3 protein after LTEP-s and SK-MES-1 cells were infected with vector-GPC3, vector-NC, sh-GPC3 or sh-GPC3 for 48 h. (**C,D**) MTT was performed to evaluate cell proliferation after LTEP-s and SK-MES-1 cells were infected with vector-GPC3, vector-NC, sh-GPC3 or sh-GPC3 for 0, 24, 48, 72 and 96 h. (**E,F**) Flow cytometry with Annexin V (FITC)/PI staining was carried out to determine cell apoptosis 48 h after LTEP-s and SK-MES-1 cells were infected with vector-GPC3, vector-NC, sh-GPC3 or sh-GPC3 (n=3, *P<0.05, **P<0.01, ***P<0.001, sh-GPC3 group vs sh-NC group; ^##^*P*<0.01, ^###^*P*<0.001, vector-GPC3 group vs vector-NC group).

Then, we explored the function of GPC3 in the proliferation and apoptosis of lung SCC LTEP-s and SK-MES-1 cells. Infection of LTEP-s and SK-MES-1 cells with lentivirus vector-GPC3 significantly increased GPC3 expression compared with cells treated with vector-NC (Figure 2A). In addition, the sh-2 vector targeting the human GPC3 gene reduced GPC3 expression by 60–70% at the mRNA level in both LTEP-s and SK-MES-1 cells (Figure 2A), hence we chose sh-2 for the following study. Consistently, sh-GPC3 significantly reduced GPC3 protein expression, while vector-GPC3 enhanced GPC3 expression (Figure 2B). In addition, up-regulation of GPC3 with vector-GPC3 transfection significantly enhanced cell growth, and down-regulation of GPC3 with sh-2 treatment obviously decreased cell growth in LTEP-s and SK-MES-1 cells (Figure 2C,D). Furthermore, up-regulation of GPC3 significantly inhibited the apoptosis of LTEP-s [from (1070 ± 65) to (660 ± 69) cells] and SK-MES-1 cells [from (1090 ± 85) to (560 ± 79) cells], and *vice versa* (Figure 2E,F). All data indicate that GPC3 accelerates the progression of lung SCC.

### Up-regulation of GPC3 increases the expression of β-catenin in lung SCC cells

**Figure 3:**
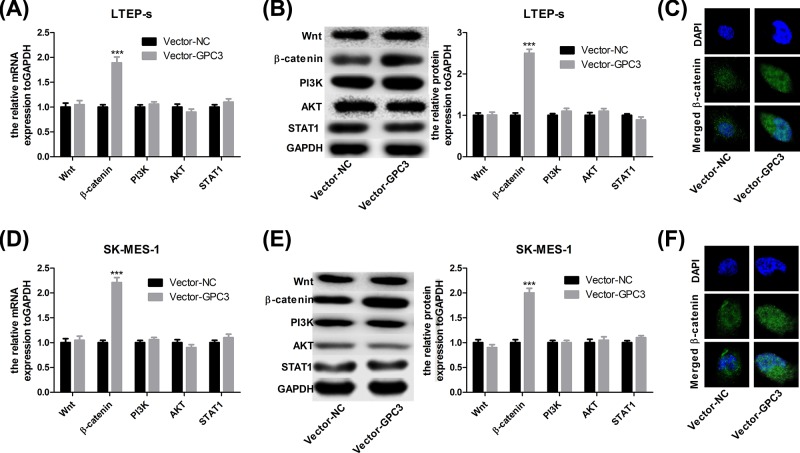
Up-regulation of GPC3 increased β-catenin expression in LTEP-s and SK-MES-1 cells Forty-eight hours after LTEP-s and SK-MES-1 cells were infected with vector-NC or vector-GPC3, cells were collected and submitted to the following assays. (**A,B**) RT-PCR and WB to analyse the mRNA and protein expression of Wnt1, β-catenin, PI3K, AKT and STAT1 in LTEP-s. (**C**) Immunofluorescence assay was used to detect the subcellular location of β-catenin protein in LTEP-s cells. (**D,E**) RT-PCR and WB were performed to analyse the mRNA and protein expression of Wnt1, β-catenin, PI3K, AKT and STAT1 in SK-MES-1 cells. (**F**) Immunofluorescence assay was used to detect the subcellular location of β-catenin protein in SK-MES-1 cells (n=3, ***P<0.001, vector-GPC3 group vs vector-NC group).

Next, we investigated the underlying mechanism of GPC3 in the progression of lung SCC. Both the mRNA and protein levels of β-catenin were increased after GPC3 was up-regulated in LTEP-s cells, whereas there was no obvious change in the expression of Wnt1, AKT, PI3K and STAT1 (Figure 3A,B), or the increased nuclear accumulation of β-catenin protein (Figure 3C). The same principle applied to SK-MES-1 cells (Figure 3D–F). These results suggest that the activation of β-catenin signalling might be involved in the progression of lung SCC mediated by GPC3 overexpression.

### GPC3 promotes the progression of lung SCC through up-regulating β-catenin expression

**Figure 4:**
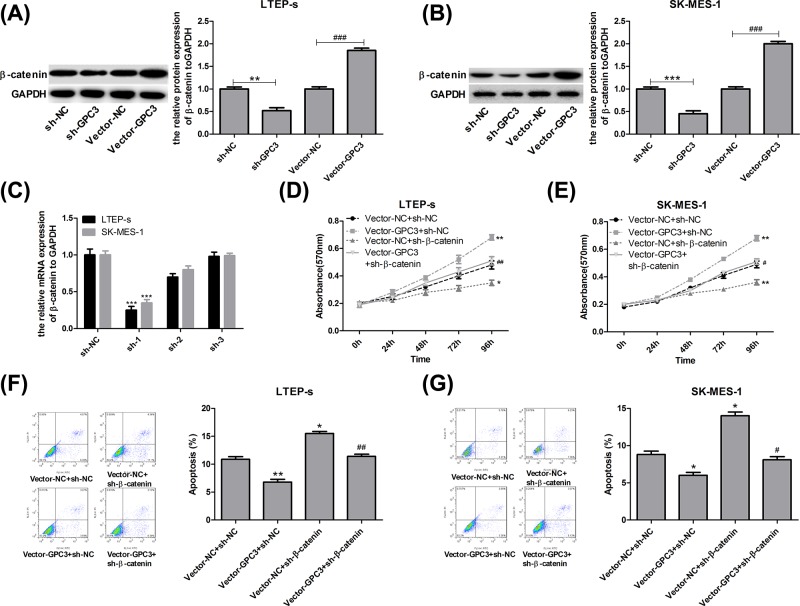
GPC3 promoted cell growth and repressed cell apoptosis through up-regulating β-catenin expression in LTEP-s and SK-MES-1 cells (**A,B**) LTEP-s and SK-MES-1 cells were infected with vector-NC or vector-GPC3, sh-NC or sh-GPC3 for 48 h, and then WB analysis was used to test the expression of β-catenin (n=3, **P<0.01, sh-GPC3 group vs sh-NC group; ^###^*P*<0.001, vector-GPC3 group vs vector-NC group). (**C**) Forty-eight hours after treatments with sh-1, sh-2 and sh-3 targeting the human β-catenin gene and sh-NC, the LTEP-s and SK-MES-1 cells were collected to evaluate the knockdown efficiency by RT-PCR (n=3, ***P<0.001, sh-1 group vs sh-NC group). Then, LTEP-s and SK-MES-1 cells were infected with vector-NC + sh-NC, vector-GPC3 + sh-NC, vector-NC + sh-β-catenin or vector-GPC3 + sh-β-catenin, followed by the following assays. (**D,E**) An MTT assay was carried out to determine cell proliferation after 0, 24, 48, 72 or 96 h of cell infection. (**F,G**) Flow cytometry with Annexin V (FITC)/PI staining was carried out to determine cell apoptosis after 48 h of cell infection (n=3, *P<0.05, **P<0.01, ***P<0.001, vector-GPC3 + sh-NC or vector-NC + sh-β-catenin group vs vector-NC + sh-NC group; ^#^*P*<0.05, ^##^*P*<0.01, ^###^*P*<0.001, vector-GPC3 + sh-β-catenin group vs vector-GPC3 + sh-NC group).

**Figure 5:**
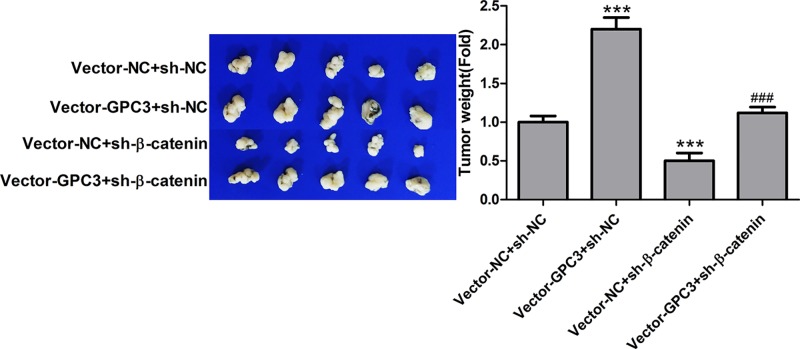
GPC3 promoted the tumorigenesis of SK-MES-1 cells through up-regulating β-catenin expression An in vivo tumour formation assay was used to evaluate the tumorigenesis of SK-MES-1 cells with stable expression of vector-NC + sh-NC, vector-GPC3 + sh-NC, vector-NC + sh-β-catenin or vector-GPC3 + sh-β-catenin. (n=5, ***P<0.001, vector-GPC3 + sh-NC or vector-NC + sh-β-catenin group vs vector-NC + sh-NC group; ^###^*P*<0.001, vector-GPC3 + sh-β-catenin group vs vector-GPC3 + sh-NC group).

Based on the above results, we further explored the role of β-catenin in GPC3-mediated lung SCC progression. Up-regulation of GPC3 significantly increased β-catenin expression, while down-regulation of GPC3 showed the opposite result in LTEP-s and SK-MES-1 cells (Figure 4A,B). sh-1 targeting the human β-catenin gene showed the best knockdown efficiency among the three shRNAs, and it reduced the expression of β-catenin by approximately 80% in LTEP-s cells and 70% in SK-MES-1 cells (Figure 4C). As the MTT assay showed, down-regulation of β-catenin significantly inhibited cell proliferation and abolished the role of GPC3 in cell growth promotion in both LTEP-s and SK-MES-1 cells (Figure 4D,E). Consistently, knockdown of β-catenin rescued GPC3 overexpression and caused cell apoptosis inhibition in both LTEP-s and SK-MES-1 cells (Figure 4F,G). Moreover, up-regulation of GPC3 increased the tumorigenesis of SK-MES-1 cells, while down-regulation of β-catenin reduced tumorigenesis, and knockdown of β-catenin weakened the tumorigenesis induced by GPC3 overexpression in SK-MES-1 cells (Figure 5). Taken together, these results demonstrate that GPC3 overexpression promotes the progression of lung SCC in a β-catenin-dependent manner.

## Discussion

Despite much progress having been made in therapy, lung SCC is still the main reason for lung cancer-related deaths [13]. Lack of clinical symptoms or effective biomarkers leads to the majority of lung SCC patients being at a more advanced stage when they are first diagnosed [14]. Therefore, it is essential to look for specific molecular markers with high sensitivity to lung cancer for early diagnosis and personalised treatment [15].

GPC3, also known as DGSX, OCI-5, MXR7, GTR2-2, SDYS, SGB, SGBS1 and SGBS, belongs to the glypican-related integral membrane proteoglycan family (GPC1–GPC6) [8] and was recently identified to play a crucial role in the molecular mechanisms by which the malignant phenotype of HCC cells are modulated. For instance, Tian et al. [16] found that the up-regulation of microRNA-133b attenuated the proliferation and invasion and increased the apoptosis of HCC cells by activating E-cadherin expression and repressing GPC3 expression. Hu et al. [17] reported that autophagy suppressed the proliferation of HCC HepG2 cells via inhibiting GPC3/Wnt/β-catenin signalling. Furthermore, GPC3 has been illustrated to be a potential biomarker candidate for HCC, acute respiratory distress syndrome and severe pneumonia [18,19]. In the present study, our results suggest that GPC3 is up-regulated in lung SCC cells and tissue samples, which then promotes cell growth and tumorigenesis and reduces cell apoptosis, demonstrating that GPC3 functions as an oncogene in lung SCC.

GPC3 functions entirely differently in various types of tumours. Specifically, GPC3 has been reported to be expressed at low levels in ovarian carcinoma [20], mesothelioma [21] and breast carcinoma [22,23] tissues compared with normal tissues. The up-regulation of GPC3 significantly inhibited the proliferation and metastasis of breast cancer cells [22], illustrating that GPC3 functions as a tumour suppressive gene in breast cancer. However, GPC3 was highly expressed in other tumours, such as HCC [24,25], Wilms’ tumour and neuroblastoma [26], hepatoblastoma [27] and melanoma [28], as well as lung SCC [12,29]. Consistent with previous studies [12,29], we also revealed that the expression of GPC3 was significantly elevated in lung SCC.

In addition, we postulated that β-catenin was closely involved in the process by which GPC3 obviously accelerated lung SCC progression. β-catenin, an irreplaceable member of the classical Wnt signalling pathway, is activated and translocates to the nucleus when Wnt proteins bind to the frizzled (Fz) receptors in a paracrine pattern [30]. It has been reported that β-catenin is frequently hyper-activated in human cancers and closely participates in the progression of many types of malignant cancers, including lung cancer [31]. For instance, Xu et al. [32] demonstrated that β-catenin was aberrantly overexpressed in 76% (189 of 262) of lung cancer tissues from patients with NSCLC. Huang et al. [33] reported that Wnt2 could promote the proliferation of NSCC cells via activating the Wnt/β-catenin pathway. Furthermore, researchers [9,17] have revealed that β-catenin signalling could also be activated by hyper-activated GPC3, thereby modulating cell proliferation. Similarly, we demonstrated that GPC3 promoted the growth and tumorigenesis and inhibited the apoptosis of lung SCC cells through up-regulating β-catenin with no obvious influence on the expression of Wnt, suggesting that there might be a direct effect of GPC3 on β-catenin.

In conclusion, the present study reveals, for the first time that GPC3 accelerates the progression of lung SCC in a β-catenin-dependent manner. Our study provides a theoretical basis for the GPC3/β-catenin axis serving as a novel therapeutic target for lung SCC.

## Abbreviations

GAPDH: glyceraldehyde-3-phosphate dehydrogenase
GPC3: Glypican-3
HCC: hepatocellular carcinoma
HRP: horseradish peroxidase
MEM: Modified Eagle Medium
NC: negative control
NSCLC: non-small cell lung cancer
RT-PCR: real-time PCR
SCC: squamous cell carcinoma
WB: Western blot
