# Expression of Concern: Glypican-3 promotes cell proliferation and tumorigenesis through up-regulation of b-catenin expression in lung squamous cell carcinoma

**DOI:** 10.1042/BSR-2018-1147_EOC

**Published:** 2024-08-14

**Keywords:** β-catenin, GPC3, lung squamous cell carcinoma, proliferation, tumorigenesis

The Editorial Office has been made aware of potential issues surrounding the scientific validity of this paper, hence has issued an expression of concern to notify readers whilst the Editorial Office investigates. It has been noted that there appears to be multiple image duplications between this paper and papers by different authors. Specifically:

- Figure 2B LTEP-s Vector-GPC3 panel which appears as Figure 4G sh-TSTA3 panel from Gao et al, 2021 (DOI: 10.3892/or.2021.8023) [retracted], and Figure 3E si panel from Zhang et al, 2019 (DOI: 10.1016/j.lfs.2019.116648)
- Figure 2B SK-MES-1 sh-NC panel which appears as Figure 2B LTEP-s vector-NC panel in this paper
- Figure 2B SK-MES-1 sh-GPC3 panel which appears as Figure 5G HO-8910 mimics panel from Zhang et al, 2019 (DOI: 10.1016/j.lfs.2019.116648)
- Figure 3F Vector-GPC3 panel which appears as Figure 2G HT-1376 OE-NC panel from Xu et al, 2020 (DOI: 10.3892/ijo.2019.4922) [retracted], and part of which appears as Figure 5G HO-8910 inhibitors DAPI panel from Zhang et al 2019 (DOI: 10.1016/j.lfs.2019.116648)
- Figure 5 parts of which appear as Figure 2I from Ruan et al, 2019 (DOI: 10.2147/cmar.s180418) [retracted], Figure 3B from Qin et al, 2019 (DOI: 10.1016/j.biopha.2018.12.007), Figure 3B from Cheng et al, 2019 (DOI: 10.1042/bsr20180906) [retracted], and Figure 7B from Sun et al, 2017 (DOI: 10.18632/oncotarget.15846)

The Editorial Office is currently in contact with the authors regarding these concerns.

